# Tracing the Link Between Narcissistic Personality Disorder and Childhood Overgratification

**DOI:** 10.7759/cureus.72638

**Published:** 2024-10-29

**Authors:** Husna Irfan Thalib, Aleena Zobairi, Sariya Khan, Mariam Abou Touk, Razan Bahkali, Sarah Alhusaynan, Suha Fatima Hussain

**Affiliations:** 1 General Medicine Practice Program and Surgery, Batterjee Medical College, Jeddah, SAU; 2 General Medicine Practice Program and Surgery, Almaarefa University, Riyadh, SAU; 3 General Medicine, Kaloji Narayana Rao University of Health Sciences, Warangal, IND

**Keywords:** childhood, developmental psychology, narcissistic personality disorder, overgratification, personality traits

## Abstract

Narcissistic personality disorder (NPD) is a psychiatric disorder that remains largely undiagnosed in modern society. Theories claim that the roots of this disorder can often be traced back to childhood experiences and parenting styles. The prevalence of NPD in the general population is estimated to be significantly high, although rates may vary widely depending on the assessment method and population being considered as a significant portion of the population remains largely unaware of this disorder or though the patient is aware of his/her condition, the bad reputation for the illness demotivates him/her from seeking professional help. This is the main reason why it is difficult to know the real prevalence of these disorders that are so frequently encountered in psychiatric practice but still largely undiagnosed. This review collects and analyzes several research studies and literature reviews found in the electronic databases. The inclusion criteria prioritize studies focusing on the effect of overgratification on the development of NPD. Empirical evidence suggests a link between childhood overgratification and the development of narcissistic traits in adulthood. However, the relationship between childhood overgratification and NPD is much more complex and is deeply influenced by various factors, such as parent-child attachment, individual nature, and cultural context. This paper aims to simplify and illustrate the complex interplay between childhood overgratification and the manifestation of NPD, thereby revealing underlying mechanisms and identifying potential interventions for treatment. This article explores the intriguing correlation between NPD and overgratification during childhood, exploring how early indulgence may sow the seeds for narcissistic personality traits in adulthood.

## Introduction and background

Narcissistic personality disorder (NPD) is a mental health condition characterized by a constant need for praise and a lack of empathy for others. Individuals with NPD often feel an exaggerated sense of self-importance. They display a pattern of grandeur and excessively fantasize about limitless success, power, and beauty. They strongly believe that they are special and unique [1]. They exploit, manipulate, or harm others to achieve their goals and display arrogant or arrogant behavior. The effects of NPD can be profoundly harmful. This can have serious consequences for the person suffering from the disorder and those around them [2]. People with NPD may have difficulty creating and maintaining healthy, meaningful relationships. This is because being self-centered and lacking empathy can drive others away. They may also face a number of difficulties in academic and professional environments. This is because the need for praise and approval can lead to strained relationships with officials or coworkers [3]. The relentless pursuit of success and power can lead to stress, constant anxiety, rage, and dissatisfaction with life. In extreme cases, a person with NPD may engage in harmful, manipulative, or exploitative behaviors, such as emotional abuse in the family [4].

Narcissism comes in many different forms. Each form has its own characteristics. Grandiose narcissism is what most people think of first. It includes a person who is arrogant, confident, and always seeking admiration. They often feel superior and take advantage of others to get what they want. Vulnerable narcissism, on the other hand, looks very different. These people are insecure and sensitive to criticism. Even if they seem shy or reserved, they also feel entitled and can be easily hurt when others receive attention. Malignant narcissism is even more dangerous because it combines narcissistic traits with aggression and a lack of empathy. Such people can be manipulative and dangerous in their quest for control. Community narcissism occurs when someone is viewed as generous or extremely selfless but they are primarily driven by a desire to be praised and accepted for their "kindness." Finally, covert narcissism, also known as "closet narcissism," is more subtle. These people may appear humble but inside they feel like they deserve more recognition and often feel misunderstood or underappreciated which commonly leads to aggressive behavior [2-5].

Childhood overindulgence refers to a parenting style that continually accepts a child's needs and desires without appropriate constraints or restrictions. This can manifest itself in many ways, such as giving too many compliments, being overprotective, or giving too much to children through material possessions or special rights. Excessive complacency can create the basis of entitlement, selfishness, and an inability to tolerate rejection or lack of attention [5]. The possible link of childhood overgratification to NPD arises from its possible role as a developmental precursor to narcissistic traits. Psychological theory suggests that excessive praise during childhood can lead to an exaggerated sense of self-importance and pride which lays the foundation for later adult narcissism [6]. This parenting style also prevents the development of empathy and caring for the feelings and emotions of others. Understanding the relationship between overgratification in children and NPD in adulthood is of critical importance in understanding the origins of NPD. This narrative review article examines the detrimental effects of parenting style and childhood environment on personality development, thereby aiding clinicians and researchers to focus on childhood interventions to address problems related to narcissistic traits and promote good mental functioning in adults [7,8].

## Review

Methods

This literature review included studies published between January 2005 and September 2024. The databases searched were Google Scholar, PubMed, Springer, and Web of Science, using keywords such as "Narcissism", "Overgratification", "Personality Development", "Parental Influence", and "Self-Perception". We included English-language studies closely aligned with the study's theme, while studies in other languages were excluded. Data extraction focused on identifying patterns, trends, and major findings, contributing to a comprehensive understanding of the relationship between overgratification and narcissistic traits.

Understanding NPS: definition and prevalence

**Figure 1:**
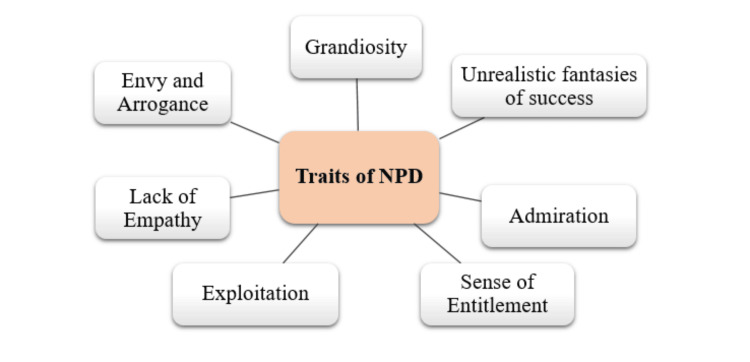
Traits of NPD as identified by DSM-5 NPD: narcissistic personality disorder; DSM-5: Diagnostic and Statistical Manual of Mental Disorders, Fifth Edition

As mentioned, NPD is a mental health condition characterized by a wide range of symptoms, such as a lack of empathy for others and a need for constant attention and praise as mentioned in Figure 1. According to the Diagnostic and Statistical Manual of Mental Disorders, Fifth Edition (DSM-5), the diagnostic criteria for NPD include the presence of at least five of the following traits, as mentioned in Figure 1 [9-16]. The prevalence of NPD in the general population is estimated to be between 1% and 6%, although rates can vary greatly depending on the assessment method and the population considered. This is because a significant portion of the population remains unaware of this disorder and therefore underdiagnosed [17]. NPD is more often diagnosed in men than women. However, this may be due to gender bias in diagnosis or the natural and traditional tendency toward overindulgence that is prevalent in men [18]. Individuals with NPD may struggle significantly to establish and maintain healthy relationships, which usually leads to emotional abuse and relationship failure. Their difficult personalities and unrealistic expectations in professional environments can lead to workplace problems with supervisors and teamwork problems [19]. Additionally, the negative effects of NPD extend beyond personal relationships and the workplace. In extreme cases, a person with NPD may engage in destructive behaviors such as manipulation, exploitation, and aggression in public. Understanding the prevalence and social impact of NPD is critical to creating widespread awareness and concerted efforts aimed at managing and treating narcissistic behavior. This will help reduce harmful impacts on individuals and communities [20].

Exploring childhood overgratification and its consequences

Overgratification refers to a parenting style characterized by excessive indulgence, where the child's needs and desires are consistently met without appropriate boundaries or limits. This type of parenting usually includes children who are overly satisfied with wealth, privilege, or attention [21]. In short, it means meeting a child's every need or desire without teaching him the basic values ​​of patience, tolerance, refusal, self-discipline, or facing rejection, especially the outburst of anger and emotional control [22]. Parental behavior plays an important role in shaping a child's personality including the child's mental, emotional, and social characteristics. When parents resort to overindulgence and overappreciation, they may subconsciously be laying the groundwork for their future adulthood, filled with a sense of entitlement, selfishness, and the inability to tolerate frustration or rejection [23]. Instead of innocently learning to deal with disappointment, these children grow up expecting immediate compliance, empathy, admiration, and appreciation from other interactions outside the family. They will become intolerant of the compliments of others in any environment. Children who grow up in an environment where every need is met strive to understand the feelings of others. This results in a lack of basic social skills such as empathy and consideration of one's own emotions [24]. This can lead to serious problems in building healthy relationships. Moreover, in extreme cases, they have no sense of responsibility because they have not been taught the importance of being rewarded for their efforts or merit [25].

The psychological effects of excessive complacency in childhood can be far more serious and deadly than anyone can imagine. There are many people struggling with feelings of dissatisfaction and emptiness. This is because they rely on external verification, constant praise, or material rewards to evaluate and satisfy inflated self-worth [26]. They may have difficulty coping with disappointments or failures in life because they have not learned to endure frustration. They may behave more impulsively or self-destructively later on in their adulthood. By realizing the severity of the impact on children's development caused by parents' attitudes and behaviors, parents or caregivers can adopt a more careful and balanced approach. This ensures long-term, healthy outcomes for children instead of fulfilling short-term wishes [27].

Different parenting styles

Parenting practices have a significant impact on a child's psychological growth influencing their behavior, emotional health, and interpersonal interactions. Different levels of responsiveness and demands are placed on the child by these four styles, authoritative, authoritarian, permissive, and neglectful, which all result in different personality and behavior outcomes [28]. The most well-rounded and successful parenting approach is generally accepted to be authoritative parenting. These parents provide structure and warmth, and at the same time, they establish boundaries and clear expectations, promote independence, and value open communication [29]. Their ability to balance firm discipline with responsiveness to their child's emotional needs helps kids grow in social skills, self-assurance, and emotional control. According to research, children who grow up in authoritative homes typically achieve better academically, have higher self-esteem, and form healthier relationships, and this shows that this parenting style may be protective against personality disorders [30]. Conversely, authoritarian parenting is defined by high expectations and poor responses. Parents set tight guidelines, demand unquestioning compliance, and frequently employ harsh methods to instill discipline. Children who receive insufficient emotional warmth and conversation may grow up to feel inadequate, have low self-esteem, and experience social anxiety. Raised in such an environment, children may develop externalizing behaviors like aggression or defiance as well as become extremely submissive or excessively rebellious. This approach is linked to the growth of people who have rigid, perfectionistic tendencies or struggle with authority [30].

Permissive parenting is characterized by minimal demands and high responsiveness. Parents who are permissive give their kids a lot of freedom and don't always enforce rules or penalties. Despite their love and support, these parents' lack of boundaries and discipline can cause their kids to lack self-control, struggle with authority, and develop an entitlement complex [31]. Since the child grows accustomed to instant gratification and may experience frustration or delayed reward, this parenting style is frequently associated with narcissistic traits. Children raised in permissive environments may also struggle with impulse control and exhibit egotistical or self-indulgent behaviors which increases the likelihood that they will grow up to have narcissistic personality traits [32]. Low responsiveness and low demands are characteristics of neglectful parenting, also referred to as uninvolved parenting. These parents frequently meet their child's physical needs but fall short of providing emotional support, direction, or discipline. They are emotionally cold and uninvolved in their child's life [33]. Neglected children may have feelings of abandonment which can cause them to struggle to form stable attachments, controlling their emotions and self-worth. Due to their tendency to look for attention and validation elsewhere, these kids are more likely to experience emotional and behavioral issues such as anxiety, depression, and possibly the emergence of narcissistic or antisocial behaviors [34]. Permissive parenting sticks out as a possible connection to NPD in the context of overgratification. One of the main characteristics of NPD in children is an exaggerated sense of self-importance and a lack of empathy for others. These traits can arise when children are continuously catered to and their desires are prioritized without boundaries or consequences. Furthermore, kids raised by indulgent or permissive parents might not learn how to deal with disappointment or frustration which could make it harder for them to deal with obstacles and setbacks as adults, since early parental influences shape a child's worldview, behavior patterns, and emotional responses [31-34].

Impact of different parenting styles on the development of narcissistic traits

Humans have an innate tendency toward narcissism. The parents can mold this natural tendency into either a maladaptive or an adaptive form. Parenting practices play a significant role in a child's narcissistic traits [35]. However, research into the topic suggested that more than one type of parenting style may be associated with children's narcissism. The research on grandiose narcissism development in children depicts two opposing styles of parenting that may foster this trait. Some research indicates that permissiveness overindulgent parenting leads to the formation of grandiose narcissism. In this context, children often remember their parents as having been convinced of their extraordinary abilities and talents, expressing them with tribute but hardly any criticism [36,37]. Such permissiveness may foster notions of entitlement and a sense of being grandiose since the child is rewarded even in the absence of any worthiness [7]. On the other hand, grandiose narcissism may also be facilitated by authoritarian parenting, characterized by the lack of emotional warmth and excessive control. Kohut's concept of "chronic frustration" further explains how a consistent lack of empathy, which could be in the absence of empathic parents, can create frustrating experiences for children severally. Such frustrating experiences inhibit the child from adequate introjection of self-objects necessary for self-esteem and regulation of emotions [38]. This results in children with feelings of inadequacy, as compensation leads to narcissistic traits when raised as teenagers [39,40]. Even more confusing is the fact that there are reports that children coming from more privileged and less constrained backgrounds can also be very grandiose. This means that even too well-meaning and indulgent parents and also very strict parents can foster some aspects of grandiose narcissism, which underlines the complexity of its development [7].

Regarding vulnerable narcissism, research shows that is connected to different parenting styles, such as inconsistent discipline and poor supervision [38]. It can also come from having overly permissive parents, as well as from parenting that focuses on warmth [35]. As stated by Baumrind, to maintain innate narcissistic tendencies in a healthy form, the authoritative parenting style is ideal. This style combines parental control and oversight with love and care. This approach enables the children to be given direction as well as emotional warmth. Children take in the experiences, where the parents are loving, caring, and responsive to their needs. This conception of psychological health helps the development of healthy narcissism [38,40]. Another critical aspect to keep in mind is the relationship between the child's age and developmental stage. Research suggests that parenting styles should adapt as the child grows to suit their developmental stage; otherwise, it could lead to narcissistic tendencies [39]. Moreover, it has been found that when ranking the influence of mothers and fathers, mothers are attributed to be more impactful than fathers, especially in cases where a single parenting style is employed. This is in harmony with the general conclusions of previous studies which confirm that it is maternal behavior that has a greater bearing on narcissism. One potential reason for this is that most mothers tend to stay with their children and are thus more likely to exert more direct effects from their parenting than fathers. Moreover, cultural constraints and pressures regarding maternal and paternal roles may help further this division as evidence points to mothers being more involved in childrearing than fathers. This may be the reason, which results in the explanation of why there is more linearity interaction between maternal parenting and narcissism than of paternal parenting which only has singular interactions with narcissism most likely in the low parenting or high indulgent settings [41].

Association between childhood overindulgence and narcissistic traits in adulthood

Childhood overindulgence is quite an important factor in the development of narcissistic traits. Overindulgence is different from "spoiling" and "permissiveness" as the latter occurs due to a child's demand, but overindulgence occurs irrespective of the child's demand. It refers to a parent providing too much time, attention, things, etc. Parenting practices have a major impact on how a child develops their personality and can show great differences in adulthood. Researchers can identify people at risk of narcissistic behavior as a child by examining the connections between parental personality and behavior [3]. An online study of 368 parents has shown that parents who use non-optimal parenting strategies, like overindulging, end up damaging their child's personality. This study also found that parents using non-optimal parenting styles themselves have narcissistic traits. As a result, we can also see how in a few possible cases narcissism can form a generational chain [42].

During adolescence and early adulthood, narcissistic qualities frequently become more noticeable as people navigate identity formation, social interactions, and goal-oriented endeavors. However, narcissistic tendencies may become less intense or more controlled as people become older and encounter obstacles in life. Confronting adulthood realities, career setbacks, relationship issues, and personal losses can cause patients with NPD to experience a decrease in over-grandiosity and attention-seeking behaviors [43]. A study was done in a California preschool to analyze the occurrence of narcissistic traits over time. Five preschool children were found with narcissistic personalities and were assessed at the ages of three, four, five, seven, 11, 14, 18, 23, and 32 to observe changes in personality and narcissism indicators over time. All of them had retained their narcissism until the age of 18, although four out of five had exhibited narcissistic personality at age 23. It can be concluded that personality and behavior at preschool foreshadow narcissistic traits in adolescence and adulthood [44].

Psychotherapeutic approaches and practical implications targeting underlying issues related to overindulgence and narcissistic pathology

Treatment of patients with NPD is quite a challenging task. In a sample of patients with the disorder, there had been a dropout rate of 63-64% from psychotherapy. This is assumed to be caused by shame, devaluation, or perfectionism [45]. Despite this, there have been studies done which show symptom reduction and improvement which proves psychotherapy to be effective. Treatment of NPD requires a long-term commitment and support from families and peers. Investigating the motives for attending therapy is crucial, as it can assist the patient in converting temporary drive into a dedication to long-term treatment. Numerous factors seem to have an impact on the motivation behind seeking therapy, including pressure from friends, family, or coworkers; feelings of unhappiness with life or the failure to achieve significant goals; an acute crisis in one's life; the worsening of a comorbid condition; and self-harm [46-49].

One of the most prevalent problems in NPD therapy is "nontreatment treatments," in which the patient receives treatment without making any progress. This is frequently caused by inverse idealization, devaluation of treatment, competitive or envious dynamics between the patient and the therapist, and imprecise goals. Patients typically show slow, steady improvements over time, but large therapeutic advances are uncommon. Developing successful treatments for NPD requires setting realistic goals. Establishing and pursuing attainable objectives that the patient is motivated to achieve protects against nontreatment and grounds the treatment in reality [48,49]. Through this treatment, the patient learns to confront and overcome self-defeating behaviors. One aspect of the treatment is being observant and understanding all the factors affecting a patient. This awareness helps the patient feel more comfortable and narrative about their condition. Throughout the course of treatment, paying curious attention to the patient's inner experiences and behaviors while maintaining a nonjudgmental attitude is helpful. Focusing on the therapeutic relationship encourages work on the capacity to resolve disagreements over perceptions, maintain connection during emotional crises, and keep an open mind during alliance, rupture, and repair cycles [50]. 

**Figure 2:**
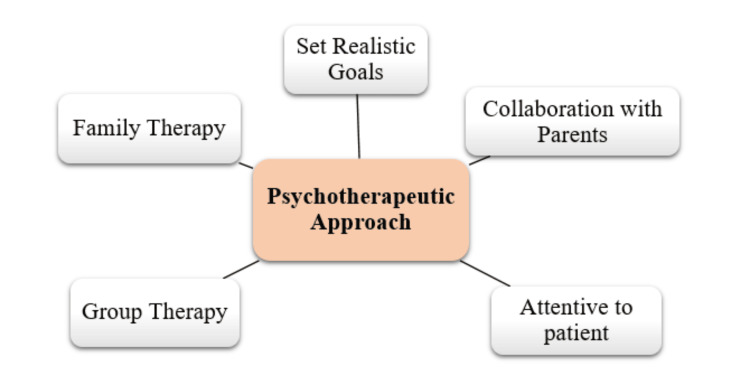
Treatment approaches for NPD NPD: narcissistic personality disorder

In order for the patient to feel connected to and trust the therapy process, a positive therapeutic relationship is necessary. Notably, the application of therapeutic strategies can impact patient-therapist relationships in a variety of ways. In order to connect and process their feelings in front of the therapist, a patient may perceive the therapist as being kind, encouraging, and trustworthy. However, there are instances when a patient may feel insecure and powerless. This internal process might result in a therapeutic alliance rupture, which must be identified and addressed [51]. The cooperation of the parents is both a strategy to increase the effectiveness of the treatment and a significant treatment goal in itself. Since parents have a profound impact on their child's development, it is crucial for them to understand how their behavior has affected their child and how they can adopt more effective parenting practices [52]. Family therapy provides an opportunity for the therapist to explore family dynamics in depth and promote a healthier parenting style that benefits everyone involved. By intervening early, family therapy can not only address current issues but also prevent future difficulties for the child. This proactive approach has been proven to be beneficial in many cases, contributing to long-term positive outcomes for both the child and the family as a whole [51,52]. Group therapy offers a supportive and validating environment that is essential for patients. In this setting, individuals have the opportunity to interact with others who share similar experiences and challenges, which can significantly enhance their interpersonal skills. Engaging with peers allows patients to practice communication, empathy, and conflict resolution in a safe space. Additionally, this group dynamic can provide valuable insights for the therapist, who can observe and evaluate each patient's relational abilities in real-time. Such observations can inform tailored interventions, ultimately fostering personal growth and development within the therapeutic process [53]. These are some of the psychotherapeutic approaches used as summarized in Figure 2.

Challenges and future directions

Current research on childhood satisfaction and NPD has several methodological limitations. Many studies on NPD in childhood have relied on retrospective self-report measures, which are based on memory bias and may not accurately include childhood experiences. Longitudinal studies often face significant challenges in following participants over long periods of time for narcissistic traits. The use of self-report measures is unreliable because individuals with NPD are less likely to report their behaviors or attitudes objectively and precisely. The study may be limited by the small sample size and lack of diverse cultural representation. This limits the generalizability of the findings. Large-scale comprehensive longitudinal studies and cross-cultural survey studies are needed to further understand the developmental trajectories of these narcissistic traits. Longitudinal studies are important to track the development of narcissistic traits from childhood through satisfaction through adulthood. Cross-cultural research is needed to understand cultural differences in child upbringing.

## Conclusions

Empirical evidence suggests a link between childhood overgratification and the development of narcissistic traits in adulthood. Theoretical studies provide insight into the developmental pathway from overgratification to NPD. However, the relationship between childhood overgratification and NPD is much more complex and is deeply influenced by various factors, such as parent-child attachment, individual nature, and cultural context. Methodological limitations of studies conducted so far include lack of credibility of retrospective self-report studies and small sample sizes. Psychotherapeutic approaches targeting underlying issues related to overgratification and narcissistic personality traits, such as psychodynamic therapy, cognitive-behavioral therapy, and schema therapy, can help individuals with NPD develop self-awareness and work on developing healthy attributes. School-based programs and community initiatives aimed at promoting healthy, balanced parenting styles and focusing on the importance of emotional learning during childhood can contribute to the prevention of narcissistic personalities. In conclusion, addressing the developmental background of NPD requires a multifaceted approach among all stakeholders including parents, researchers, psychologists, and clinicians. By addressing the previously mentioned challenges and focusing on prospects, there is hope for a more comprehensive understanding of NPD and its prevention and treatment.
